# Pupillary light reflex to light inside the natural blind spot

**DOI:** 10.1038/srep11862

**Published:** 2015-06-26

**Authors:** Kentaro Miyamoto, Ikuya Murakami

**Affiliations:** 1 Department of Life Sciences, The University of Tokyo, Tokyo 153-8902, Japan; 2 Department of Physiology, The University of Tokyo School of Medicine, Tokyo 113-0033, Japan; 3 Japan Society for the Promotion of Science, Tokyo 102-8472, Japan; 4 Department of Psychology, The University of Tokyo, Tokyo 113-0033, Japan

## Abstract

When a light stimulus covers the human natural blind spot (BS), perceptual filling-in corrects for the missing information inside the BS. Here, we examined whether a filled-in surface of light perceived inside the BS affects the size of the short-latency pupillary light reflex (PLR), a pupil response mediated by a subcortical pathway for unconscious vision. The PLR was not induced by a red surface that was physically absent but perceptually filled-in inside the BS in the presence of a red ring surrounding it. However, a white large disk covering the BS unexpectedly induced a larger PLR than a white ring surrounding the BS border did, even though these two stimuli must be equivalent for the visual system, and trial-by-trial percepts did not predict PLR size. These results suggest that some physiological mechanism, presumably the retinal cells containing the photopigment melanopsin, receives the light projected inside the BS and enhances PLR.

In the human visual system, the retina of each eye has a rod/cone photoreceptor-free region where ganglion-cell axons converge and leave the eye as the optic nerve. This structure, called the optic disk, corresponds to a BS in the visual field. However, with monocular viewing, one does not see a distinct hole there but a seamless visual scene continuing in and around the BS. Thus, when the area surrounding the BS is covered by a uniform red colour, the same red colour is perceived to fill in the BS like a uniform surface. This phenomenon is called perceptual filling-in^1^,^2^,^3^ and is attributed to a cortical process in an early visual processing stage: neurons located in layer 6 of the monkey V1 are known to respond to large stimuli covering the BS^4^,^5^,^6^. Perceptually filled-in motion in the BS is processed through a motion-processing pathway in the same manner as real motion^7^. However, it is unknown whether perceptually filled-in light inside the BS affects the magnitude of short-latency PLR, a pupillary response mediated by a subcortical pathway for unconscious vision.

Previous studies have suggested that the neural substrate responsible for the PLR is distinct from, but influenced by, the cortical pathway for visual processing. In a study of patients with visual loss, an elevation of the PLR threshold rather than a loss of pupillary functions was reported in the blind region of the visual field^8^. In another study, the pupils of patients with deficits in cortical visual functions responded exclusively to a change in light intensity, and not to a change in spatial pattern^9^. To what extent cortical processes influence the simple light reflex of the pupil remains a controversy.

Our rationale was based on the well-known fact that the pupil constricts more in response to a bright stimulus of a larger size^10^,^11^. This fact has long been known, but no study has assessed whether it is the physical or perceived size of the stimulus that determines the amount of pupillary contraction. In the present study, we stimulated the border of the BS and examined the magnitude of the PLR. The perceived size of this stimulus was larger than the actual stimulation because perceptual filling-in occurred instantaneously and a large uniform surface covering the BS was perceived even though actual light stimulation was confined to an annular region surrounding the BS.

## Results

### Relation between the PLR and perceptual filling-in

**Figure 1:**
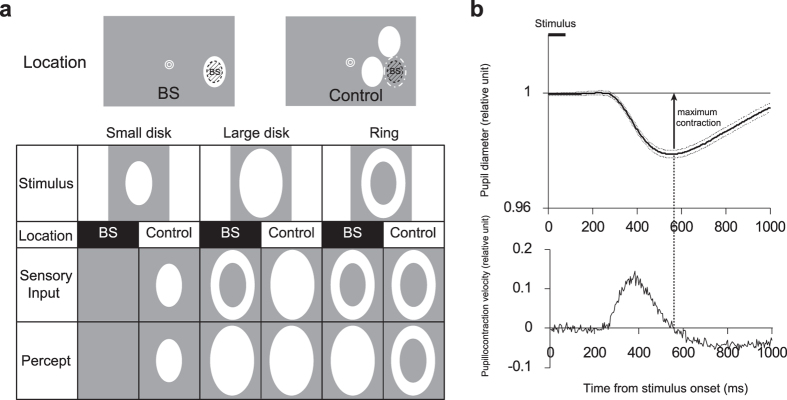
Stimuli schematics and a typical time course of pupillary light reflex (PLR). (**a**) Stimuli schematics. The small disk, large disk, and ring were displayed either at the blind spot (BS) (test condition) or adjacent to the BS (control condition). The shaded regions in the insets indicate the BS. If the area of stimulated photoreceptors determines the PLR magnitude, the large disk presented outside the BS (i.e., in the control condition) is expected to induce the greatest PLR. If the perceived area determines the PLR, the large disk and ring presented at the BS (test condition) and the large disk presented outside the BS (control condition) should all induce equally large PLR. (**b**) Typical time course of pupil diameter (upper panel) and its velocity (lower panel) measured in the present study. In each trial, the pupil diameter was normalized to the average diameter in the 500 ms prior to stimulus onset. The average pupil diameter change from the baseline ± 100 ms around the time of the maximum contraction was considered the PLR size (relative units) for each trial.

To examine whether perceptual filling-in influences the PLR, we presented three kinds of stimuli to the BS or its vicinity (Fig. 1a). The “small disk” was the same as the estimated BS shape. The “large disk” was twice as large as the BS. The “ring” was created by subtracting the small disk from the large disk. When the area of the BS was unity, the retinal areas with rod/cone photoreceptors actually stimulated by the small disk, large disk, and ring were 0, 1, and 1, respectively, when the BS was located in the middle of these stimuli. However, the large disk and the ring were perceived as a surface with an area of 2 because the missing information inside the BS was perceptually completed. Therefore, two alternative predictions were possible. If the PLR depended on the stimulated area of the photoreceptor layer of the retina, the relative PLR values should have a pattern of {0, 1, 1, 2, 1, 1} for the six conditions listed in Fig. 1a from left to right. If the PLR depended on the perceived area of light, the PLR pattern should be more similar to {0, 1, 2, 2, 2, 1}.

**Figure 2:**
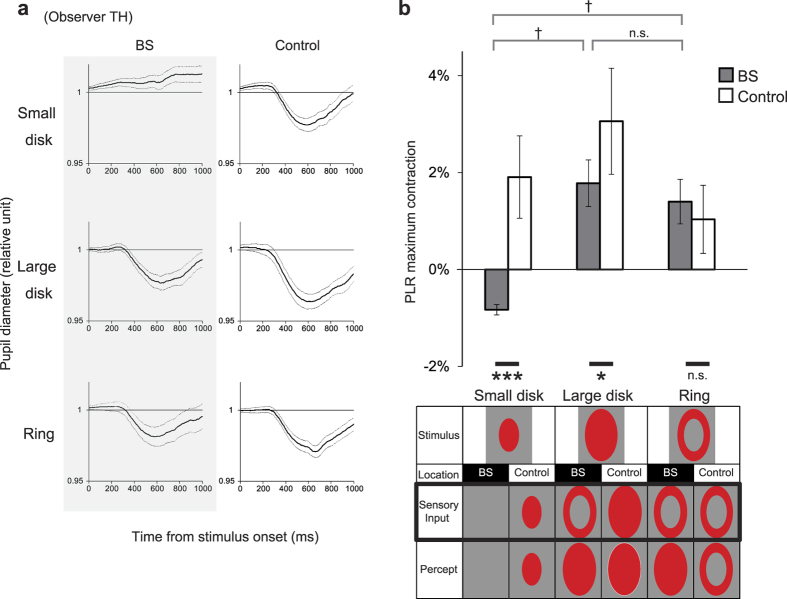
Pupillary light reflex (PLR) in response to red light stimuli with perceptual filling-in. (**a**) Typical time course (mean ± 1 s.e.m.) of pupil diameter in response to red light stimulation in a single observer. (**b**) PLR size (mean ± 1 s.e.m.) in all stimulus conditions for red light [N = 5 observers]. *p < 0.05, ***p < 0.005, †p < 0.0001 (Bonferroni-corrected).

The time course of pupil constriction was consistent with the typical short-latency PLR in each of the six conditions in each observer (Fig. 2a). To examine the effect of illusory light inside the BS on the PLR, the maximum pupillary contraction (see Fig. 1b) was compared across conditions (Fig. 2b). Initially, we used a red colour to drive the PLR, as the red phosphor of the CRT monitor had a spectrally confined irradiance and the shortest decay time constant of all phosphors. Two-way repeated-measures ANOVA of position (BS, control) × shape (small disk, large disk, and ring) identified a significant interaction between position and shape (F(2,8) = 14.5, p = 0.02). At the BS position, the small disk did not decrease the pupil diameter from the baseline. This is a reasonable result, as the small disk occupied the same region as the estimated BS, so no rod/cone photoreceptor activation was expected. Rather, the pupil diameter increased slightly, and this pupil dilation developed gradually without clear latency locked to the stimulus onset (see Fig. 2a, “small disk” at “BS”). This effect could be attributed to autonomic responses mediated by arousal signals conveyed by sympathetic nerves, which might have been activated because of difficulties performing the attentional fixation task before the stimulus presentation^12^. At the control position, when the whole stimulus was presented outside the BS, the large disk induced larger PLR than the small disk and the ring (large disk vs. small disk: t(4) = 3.2, p < 0.0001; large disk vs. ring: t(4) = 5.7, p < 0.0001, Bonferroni-corrected). This is consistent with the fact that the PLR becomes greater as the stimulated area increases^10^. Importantly, the magnitude of the PLR for the large disk at the BS position was smaller than that for the same large disk at the control position (F(1,12) = 5.0, p = 0.04), and was comparable with that for the small disk and the ring at the control position. On the other hand, the magnitude of the PLR was similar for the ring at the BS position and the ring at the control position (F(1,12) = 1.7, p = 0.20). These results showed a pattern of {0, 1, 1, 2, 1, 1} in relative terms and were compatible with the hypothesis that the PLR depends on the physical area of rod/cone photoreceptor stimulation, not the perceived area.

**Figure 3:**
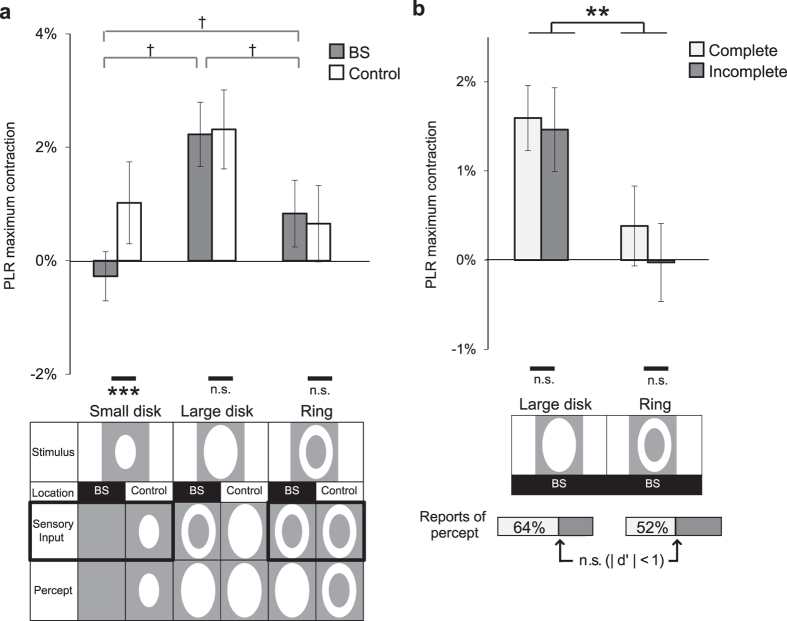
Pupillary light reflex (PLR) in response to white light stimuli with perceptual filling-in. (**a**) PLR size (mean ± 1 s.e.m.) in all stimulus conditions for white light [N = 10 observers; 5 in the meridian-control condition and 5 in the eccentricity-control condition]. ***p < 0.005, †p < 0.0001 (Bonferroni-corrected). (**b**) PLR size (mean ± 1 s.e.m.) as a function of presented stimulus shape (large disk or ring) and actual percept (complete or incomplete disk) [N = 5 observers]. The bars at the bottom indicate the relative frequency of the percept. **p < 0.01.

To determine whether the above findings could be generalized, we next tested white stimuli with more broadband spectra; all red, green, and blue phosphors of the CRT monitor were used to display light stimuli. Again, two-way repeated-measures ANOVA of position (BS, control) × shape (small disk, large disk, and ring) identified a significant interaction between position and shape (F(2,18) = 5.17, p = 0.01). At the control position, the results for the white stimuli were the same as for the red stimuli (Fig. 3a), i.e., the large disk outside the BS induced a larger PLR than did the small disk and the ring (large disk vs. small disk: t(9) = 6.7, p < 0.0001; large disk vs. ring: t(9) = 7.8, p < 0.0001, Bonferroni-corrected). At the BS position, the ring stimulus induced the same moderate PLR as did the small disk and as the ring positioned outside the BS, consistent with the results for the red stimuli. However, the PLR for the white large disk at the BS position was significantly greater than that for the ring at the same position (t(9) = 10.1, p < 0.0001) and was equivalent to the PLR for the large disk at the control position (F(1,27) = 0.37, p = 0.54). These results were puzzling, because the large disk and ring on the BS were expected to yield the same rod/cone photoreceptor activation and both should have been perceived as a large disk. The only physical difference between the two stimuli was the light area illuminating the BS; however, no PLR was induced by illumination of this area only, i.e., by the small disk alone at the BS position.

Why did such a strong PLR occur in response to the large white disk at the BS position? To test the possibility that observers somehow saw some differences between the large disk and ring on the BS, we randomly presented the large disk or ring on the BS in each trial and asked each observer to judge whether the stimulus was a complete disk. The signal detection theory detectability index (d’) was less than unity for each observer, indicating that observers could not reliably discriminate between the two stimuli. We sorted pupillary contraction depending on which shape was reported in each trial and compared the pupil diameter across all combinations of stimulus shapes and percepts (Fig. 3b). A two-way ANOVA of shape (large disk, ring) and percept (complete disk, incomplete disk) revealed a main effect of stimulus shape (F(1,4) = 23.9, p = 0.008) but no main effect of percept (F(1,4) = 2.1, p = 0.22), with no interaction (F(1,4) = 0.01, p = 0.91). Therefore, regardless of the perception, the large disk induced a greater PLR than did the ring at the BS position.

### Critical determinant of the short-latency PLR

The stimuli were presented peripherally (at approximately 15 degrees eccentricity) around the BS of the right eye, and were confined within a narrow area that was not larger than twice the size of the BS. To avoid the extinction of perception of peripheral stimuli due to Troxler’s fading^13^,^14^, the stimuli were presented for only 80 ms, which is the minimum duration required for temporal summation of visual stimulation and thus for the PLR to occur^15^. To prevent the light projected on the optic disk from getting scattered / reflected back into the eyeball to a biologically substantial extent, the luminance was set lower than that in previous reports of the properties of the PLR^11^. Before experiments it was verbally confirmed that observers could never perceive the small disk stimulus presented within the BS. Because of these spatial and temporal constraints of stimulus presentation, the magnitude of the PLR was naturally smaller than in previous reports^11^. However, the maximum velocity of pupil constriction was typically 10–20%/s in the present study (see Fig. 1b and Supplementary Fig. S1), and was comparable with previous reports of the short-latency PLR in response to bright stimuli^11^. In addition, the main results on the differential amount of PLR across conditions (Fig. 3a) were reproduced when the PLR was evaluated by a different criterion, using the maximum velocity of the pupil constriction (Supplementary Fig. S1). A one-way repeated-measures ANOVA revealed a significant main effect of stimulus condition (F(4,36) = 39.3, p < 0.0001), and a multiple comparison analysis confirmed that maximum velocity was greater for the white large disk than for the other three stimuli, whether the large disk was located at the BS position or the control position (p < 0.0001, Bonferroni-corrected). Therefore, the transient pupil responses we observed can be considered typical short-latency PLR to bright stimuli.

Pupil contraction began at 293 ± 20 ms (mean ± 1 s.d.) after stimulus onset for white light and 313 ± 14 ms after stimulus onset for red light, with no significant difference across conditions (white: F(4,36) = 1.53, p = 0.21; red: F(4,16) = 0.35, p = 0.83). The short latency of the response in all conditions also indicates that the reflex we observed is the short-latency PLR observed in response to bright light in previous reports^11^. The magnitude of the PLR for achromatic stimuli is proportionate to the increment of luminance. The pupil reacts transiently to changes in colour, even if the mean luminance is unchanged^16^. In the present study, the luminance of white and red stimuli was adjusted to result in comparable amounts of PLR. The maximum pupillary contraction for a large disk outside the BS was 2.3 ± 0.6% for a white disk and 3.0 ± 1.0% for a red disk (p = 0.58, t-test). These well-ordered features indicate that the reflex observed here is mediated by a common visual pathway of short-latency PLR for both luminance and colour.

Changes in the spatial patterns of the stimulus without an increment in average luminance also induce pupil responses^17^. The size of pupillary contraction in response to gratings changes systematically with spatial frequency and shows a response curve similar to the observer’s contrast sensitivity function^9^,^18^. To test the possibility that a change in spatial pattern contributed to the pupil responses observed here, we measured the amount of PLR when the same large disk was presented for 80 ms at or near the BS but with *decrement* in luminance. A two-way repeated-measures ANOVA of position (BS, control) × luminance (increment, decrement) revealed a significant main effect of luminance (F(1,2) = 9.13 × 10^2^, p = 0.001) but no significant main effect of position (F(1,2) = 0.46, p = 0.56) and no interaction (F(1,2) = 9.40, p = 0.09); as a result, no PLR occurred when luminance was *decreased* (Supplementary Fig. S2). Therefore, the PLR in the present study was indeed evoked by luminance increment and/or chromatic change rather than by any other visual attributes processed in higher cortical areas.

However, the big question remains as to why a white large disk at the BS position induced a larger PLR than a white ring at the BS position even though the retinal input at the level of the photoreceptor layer must have been the same between these conditions—without rod/cone photoreceptors to get differentially modulated by the large disk and ring, there would be no way to know which stimulus is being presented. It is unlikely that light scatter from the optic disk plays a role in the difference in the PLR, because illumination inside the BS alone was completely invisible to our observers and also induced no PLR (Figs 2 and 3).

### Influences of unseen light at the BS on the PLR

**Figure 4:**
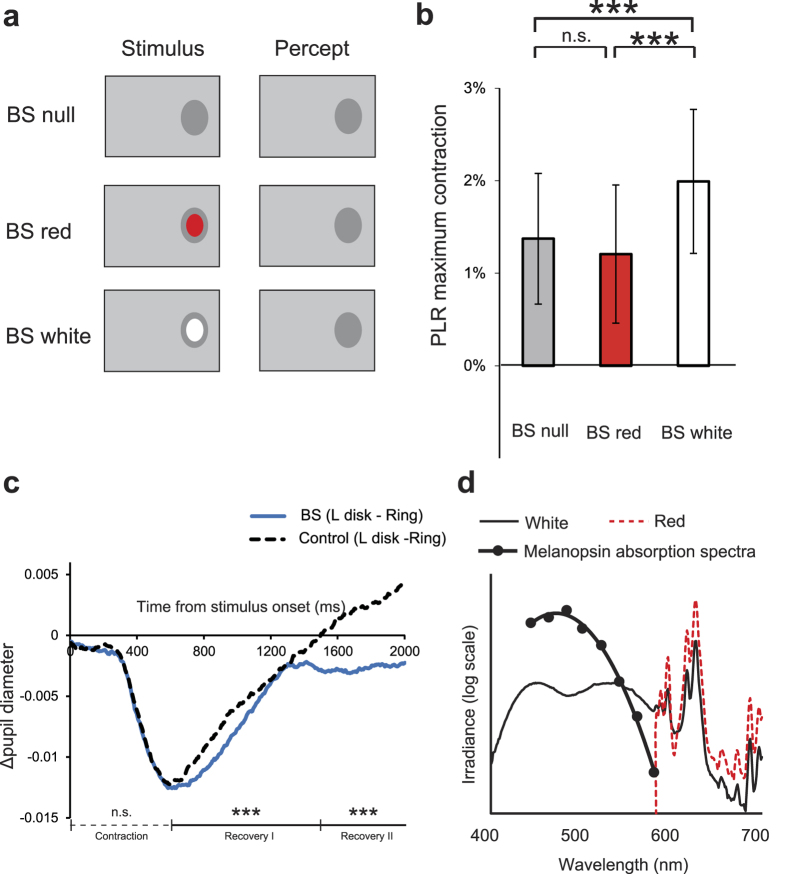
Pupillary light reflex (PLR) in response to light stimulation inside the blind spot (BS) without perceptual filling-in. (**a**) Stimulus schematics. Under all conditions, the background luminance increased transiently and yielded a perception of a uniform screen-wide grey, except within the large-disk-sized area around the BS. Under the “BS white” and “BS red” conditions, a white or red small disks was presented inside the BS simultaneously with the background change. (**b**) PLR size (mean ± 1 s.e.m.) across conditions [N = 4 observers]. *** p < 0.005 (Bonferroni-corrected). (**c**) Difference in the time course of pupil diameter (Δdiameter) between presentation of the large disk and presentation of the ring at the BS (blue trace) and outside the BS (black dotted trace). ***p < 0.005; n.s. p = 0.70 (ANOVA). (**d**) Irradiance spectra of the CRT monitor used. The white and red light spectra are plotted against wavelength. The melanopsin absorption spectra are overlaid, adapted from Gamlin *et al.*^22^.

To test whether the difference in the magnitude of the PLR between the red and white stimuli was repeatable without perceptual filling-in, we created a new stimulus. At the same time as the small disk stimulus was presented inside the BS, the background luminance was increased transiently very slightly, except for a small region around the BS that corresponded to the region of the ring in the original experiment (Fig. 4a). Therefore, whereas the large disk in the original experiment was equivalent to the simultaneous stimulus onset of a small disk inside the BS and a *ring* surrounding the BS, the new stimulus was equivalent to the simultaneous stimulus onset of the small disk inside the BS and *all the background but the ring region*. The background luminance increment was adjusted to be just enough to induce moderate pupil contraction. We examined whether a red small disk (“BS red”), a white small disk (“BS white”), or no disk (“BS null”) inside the BS could modulate the PLR in response to the same background luminance change. Importantly, these small disks could not be perceived because they were presented inside the BS, and these three conditions were indistinguishable for observers.

A one-way repeated-measures ANOVA of stimulus condition (BS null, BS red, BS white) showed a significant main effect of stimulus condition (F(2,6) = 73.1, p = 0.0001), and a multiple comparison analysis clarified that the white disk yielded a greater PLR than no disk (t(3) = 9.0, p = 0.0003, Bonferroni-corrected), but that the red disk yielded a similar PLR to no disk (t(3) = 2.3, p = 0.15, Bonferroni-corrected). Therefore, presenting the white disk inside the BS was insufficient to activate the PLR by itself (Fig. 3a), but was sufficient to enhance the PLR to other light delivered outside the BS (Fig. 4b). This was the case with or without perceptual filling-in. It follows from these findings that perceptual filling-in is unrelated to the magnitude of the PLR and white light inside the BS must be detected in some way and used in the underlying PLR mechanism.

To evaluate the possible contribution of white light illumination inside the BS, we calculated the difference in time course of pupil diameter (Δdiameter) in response to a large disk and a ring at the BS (Fig. 4c; BS, blue trace). Compared with white light outside the BS (Fig. 4c; Control, black dotted trace), a more sluggish component of pupil responses attributable to reaction to white light inside the BS was observed (latency of peak Δdiameter relative to stimulus onset: BS: 760 ± 90 ms, Control: 566 ± 36 ms; BS vs. Control: p = 0.04, paired t-test). The time course of the recovery of Δdiameter from the peak contraction was also significantly different for white light presented inside and outside the BS, whereas the time course of Δdiameter in the contraction period was similar. The processes for pupil responses to light inside the BS seem qualitatively different from those to light outside the BS, since responses to light inside the BS have a more sluggish recovery profile than a typical short-latency PLR’s.

### Blue preference of the enhancement of the PLR

To summarize the above findings, white illumination inside the BS enhances a short-latency PLR that was elicited by another illumination outside the BS, whereas red illumination inside the BS is not effective. We further examined how the increase in PLR caused by light stimulation inside the BS depends on wavelength spectral distribution, to address the possibility that melanopsin, a newly found photopigment, plays a critical role (see Discussion). Melanopsin has been found recently in special retinal ganglion cells, called intrinsically photosensitive retinal ganglion cells (ipRGCs), whose activation is deemed essential for PLR^19^.

**Figure 5:**
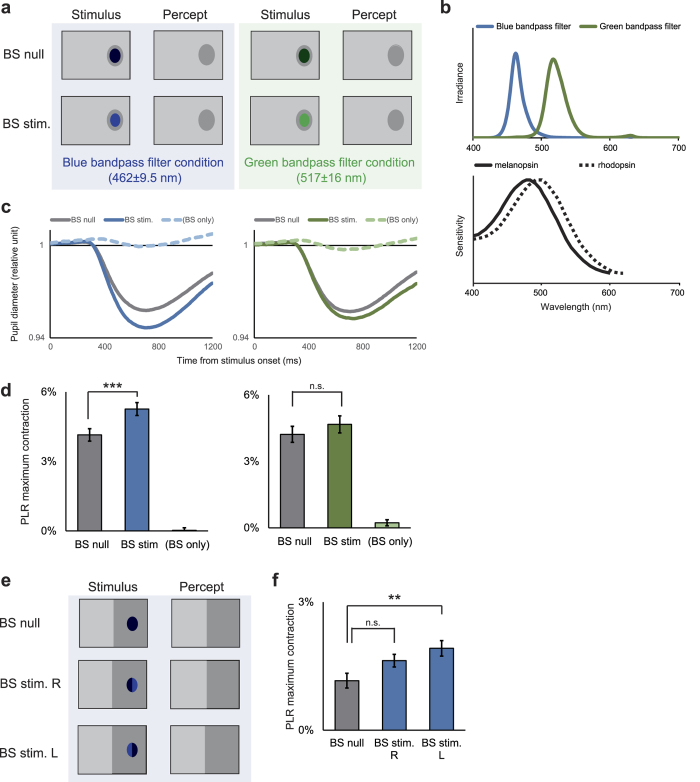
Pupillary light reflex (PLR) in response to light stimulation inside the blind spot (BS) with restriction of wavelength spectral distribution. (**a**) Stimulus schematics. In both the “BS null” and “BS stim.” conditions, the background luminance increased transiently and yielded a perception of a uniform screen-wide grey field, except within the large-disk-sized area around the BS. In the “BS stim.” condition, a green or blue disk was presented inside the BS simultaneously with the background change. In the “BS only” condition, a green or blue disk was presented inside the BS without any background change. (**b**) Wavelength spectral distribution of the blue (peak wavelength: 462 nm, full width at half maximum [FWHM]: 9.5 nm) and green (peak wavelength: 517 nm, FWHM: 16 nm) stimuli (upper panel) and photosensitivity functions of melanopsin (solid line) and rhodopsin (dashed line) adopted from Hatori and Panda^34^ (lower panel). (**c**) Average time course of pupil diameter in the experiment using the blue disk (left panel) and green disk (right panel). (**d**) PLR size (mean ± 1 s.e.m.) in each condition in the experiment using the blue disk (left panel) and green disk (right panel). ***p < 0.005 (Bonferroni-corrected). In both experiments, the PLR sizes in the “BS null” and “BS stim.” conditions were significantly greater than the PLR size in the “BS only” condition (p < 10^−5^, Bonferroni-corrected). (**e**) Stimulus schematics. In all conditions, the background luminance in the left visual hemifield increased transiently and yielded a perception of light grey field in the left visual hemifield. Under the “BS stim. R” condition, the right half of the BS was illuminated by the blue light simultaneously with the background change. Under the “BS stim. L” condition, the left half of the BS was illuminated by the blue light simultaneously with the background change. (**f**) PLR size (mean ± 1 s.e.m.) in each condition. **p < 0.01 (Bonferroni-corrected).

We conducted experiments identical to that illustrated in Fig. 4a, but using a small disk stimulus in blue or green (Fig. 5a,b). The blue light was chosen so as to be less optimal for rod photoreceptors because melanopsin should be activated more than rhodopsin, whereas the green light was chosen so as to drive rhodopsin better than melanopsin. The luminance of each of these stimuli was adjusted so that they would induce a PLR of comparable size if they were presented outside the BS at the same eccentricity (Supplementary Fig. S3). The results demonstrated that blue-light stimulation inside the BS increased the magnitude of the short-latency PLR triggered by light stimulation outside the BS (BS stim. vs. BS null: t(72) = 2.90, p = 0.003, Bonferroni-corrected), while the green-light stimulation did not (BS stim. vs. BS null: t(74) = 0.85, p = 0.92, Bonferroni-corrected) (Fig. 5c,d). To minimize the possibility that the light scattered out of the optic disk to a significant degree, we also shrank the small disk to 80% and examined whether the above pattern of results was replicated. Again, only the blue light (BS stim. vs. BS null: t(73) = 3.99, p = 3.3 × 10^−4^, Bonferroni-corrected), and not the green light (BS stim. vs. BS null: t(81) = −0.05, p = 1.0 , Bonferroni-corrected), was able to increase the PLR (Supplementary Fig. S4).

Finally, to investigate the spatial specificity of the effect brought by the interaction between light stimulations outside and inside the BS, we confined the region of the background luminance increase only within the left visual hemifield, and examined if illuminating the left or right half of the BS by the blue light enhances the short-latency PLR (Fig. 5e). The results demonstrated that stimulation in the left half, but not in the right half, of the BS increased the magnitude of PLR compared with the condition in which no stimulation was applied to the BS (BS stim. L vs. BS null: t(100) = 2.92, p = 0.008, Bonferroni-corrected; BS stim. R vs. BS null: t(120) = 2.05, p = 0.11, Bonferroni-corrected) (Fig. 5f). The left half of the BS corresponds to the optic disk portion in which ipRGC axons from the left visual hemifield converge before getting out of the eye.

## Discussion

The present study demonstrated that perceptual filling-in of the BS does not affect the short-latency PLR. The reflex increases with increasing stimulus size, but it is the physical stimulus size received by the retina, not the perceived stimulus size, that governs the amount of reflex. Indeed, trial-by-trial percepts of the presented stimuli did not predict PLR size. Therefore, no interplay occurs between the subcortical mechanism of the short-latency PLR and cortical visual processing involved in perceptual filling-in in the BS. In an electrophysiological study of monkeys^5^, V1 neurons representing the inside of the BS responded to light stimuli outside the BS with a latency 12 ms longer than that of neurons receiving direct retinal afferent inputs. If these V1 neurons participate in the process of perceptual filling-in, it might require some extra time for visual information to take an indirect route before arriving at the cortical representation of the inside of the BS. It may be a reasonable design principle that subcortical visual circuits devoted to quick pupillary reactions to abrupt light changes are driven directly by physical light information rather than waiting for the time-consuming cortical process for perceptual filling-in. On the other hand, image-forming information processing in the cortex is known to affect pupil responses with longer latencies^9^.

Additionally, the present study suggests possible existence of a physiological mechanism that receives light inside the BS and does not trigger but does enhance the PLR. One possibility is that the BS area was overestimated. In this case, the small disk, which was the same shape as the estimated BS, would effectively have stimulated photoreceptors located within the region inside the estimated BS but outside the true BS. In peripheral viewing, sensitivity to red light might be reduced compared with that to white light, which might explain why red light was ineffective. Another possibility is that the small disk, which was supposed to be confined to the BS, unexpectedly scattered a fraction of light out of the BS into the eyeball. However, in either case, observers should have been able to detect light spilled out from the BS. Actually, observers were not able to detect stimuli that were nominally inside the BS, and they could not discriminate between the complete large disk and the ring (Fig. 3b). Moreover, illumination inside the BS alone evoked no PLR (Figs 3a and 5a). Thus, although these possibilities cannot be completely rejected, they seem unlikely.

Rods are dominantly distributed on the retina around the BS at 10–15 degrees eccentricity from the fovea. Indeed, rod photoreceptors are less sensitive to red stimuli than to white stimuli that also contain shorter wavelengths of light. However, in the present study, the luminance of the red and white small disks presented adjacent to the BS were adjusted such that they should induce a comparable PLR. Even though both the red and white lights presented outside the optic disk induced an equal amount of PLR under these control conditions, only the white light but not the red light inside the BS contributed to an increase in PLR magnitude. It is unlikely that light scattering accounts for the differences because neither white nor red light presented within the BS induced PLR by itself. Also, a small disk stimulus within the BS whose spectra were preferable for rods did not significantly increase the magnitude of PLR, whereas a small disk with its spectral distribution shifted toward shorter wavelengths, which were less optimal for rods, significantly increased the magnitude of PLR (Fig. 5a–d). Even when the size of the small disk was shrunk to 80%, the same pattern of results was obtained (Supplementary Fig. S4). Therefore, our observation is not accounted for by scattered light which might be received by rods outside the BS. A recent study showed that cones uniquely sensitive to shorter wavelengths (S-cones) were better able to drive the PLR in response to gradual changes in illuminance than cones sensitive to medium wavelengths (M-cones)^20^. In this context, a possible explanation for the results shown in Fig. 5a−d is that a fraction of the light is scattered out of the optic disk, and that the scattered blue light predominantly received by S-cones outside the BS makes a greater contribution to increasing the PLR than the scattered green light predominantly received by M-cones. However, the luminances of the green and blue stimuli were adjusted to yield an equivalent PLR when presented on a retinal region adjacent to the BS. Therefore, if it existed at all, scattered light would have an equivalent effect on the PLR if mediated mainly by S-cones or by M-cones. Moreover, when the PLR was triggered by increment of the background luminance only within the left visual hemifield, stimulation of the left half of the BS (but not of the right half) increased the magnitude of the PLR (Fig. 5e,f). This asymmetrical result cannot be explained by light scattering. Therefore, it is unlikely that S-cones outside the BS contribute to the observed increase in the PLR by receiving scattered light reflected from the optic disk.

We propose the contribution of melanopsin expressed in ipRGCs, which are presumably involved in the regulation of circadian rhythms, PLR, and other non-visual responses to light^19^,^21^,^22^,^23^, and possibly in brightness discrimination in humans^24^. Strong illumination delivered to ipRGCs induces action potentials in these cells, even in the absence of visual inputs via cones or rods^19^,^25^. Activities of melanopsin-containing ipRGCs induce a longer-latency PLR than that caused by rod and cone activation when ipRGCs alone are specifically illuminated^26^. However, it is unknown how phototransduction by ipRGCs might contribute to the amount of PLR triggered by rod and cone activation. Some anatomical studies have identified immunostained melanopsin not only in the ipRGC somas, but also along the plasma membranes in the optic-fibre and ganglion-cell layers^27^. Axons containing melanopsin have also been observed^27^. These axons extend across the retinal surface and converge at the optic disk, the retinal region corresponding to the BS, such that melanopsin is expressed wherever incoming light hits the retina (inside and outside the BS) but is not expressed after the optic nerve leaves the retina and enters the brain^27^,^28^,^29^. Moreover, the light-absorption spectral profile of melanopsin peaks at 482 nm. The spectral sensitivity of the sustained pupillary response after light stimulation closely matches the melanopsin absorption spectra^22^.

For an individual lacking rods/cones, Gooley *et al.*^25^ systematically examined a contribution of ipRGCs to long-latency PLR that was distinct from typical short-latency PLR induced by rod/cone stimulation. In the present study, we focused on the contribution of melanopsin in the BS where no rods/cones exist even in normal observers, to the PLR, just as Gooley *et al.*^25^ focused on the contribution of melanopsin to the PLR in a patient without rods and cones. However, whereas Gooley *et al.*^25^ assessed the effects of melanopsin itself on the PLR in the absence of rods/cones in the patient, the present study examined the effect of interactions between ipRGCs and rods/cones on the PLR in normally sighted individuals. This was only possible if we stimulated retinal regions outside the BS, where rods/cones are dominantly distributed, and examined the effects of simultaneous light stimulation inside the BS, where melanopsin alone (not rods/cones) is expressed within the passing axons of ipRGCs^27^, within a normally sighted individual. Our results indicated that, while it did not trigger a short-latency PLR on its own, light within the BS (presumably received by melanopsin only) enhances the magnitude of a short-latency PLR only when it has already been triggered by rods/cones. The stimulus conditions in the present study were optimal to observe this effect independent of any other PLR components that may be triggered by melanopsin alone, as the PLR triggered by rods/cones can reach its maximum sufficiently before the latency of the PLR driven by melanopsin alone (typically 7.2 s)^25^.

Figure 4d shows the irradiance spectra of the red and white stimuli. A known profile of the melanopsin absorption spectra^22^ is overlaid. The figure clearly shows that melanopsin is blind to the red stimuli we used, whereas the white stimuli contained wavelengths that overlap with the melanopsin light-absorption peak. Melanopsin-containing ipRGCs receive inputs from rods/cones, and activation of ipRGCs is deemed essential for a PLR^19^. However, there remains a question about the physiological relation between input from rods/cones to ipRGCs and activation of melanopsin photopigment expressed along the axons and cell bodies of the ipRGCs. Based on these data, we speculate that melanopsin expressed along axons that pass through the optic disk (i.e., inside the BS) might absorb the light we delivered within the BS and increase the excitability of ipRGCs, eventually leading to greater excitation in the light reflex pathway. Melanopsin itself does not induce a PLR with a latency as short as 300 ms, but activation of melanopsin expressed along axons might increase the firing probability of neurons involved in the PLR when these cells are already activated by retinal illumination outside the BS. Actually, the PLR onset latency was fast and similar across all conditions we investigated. These observations ensure that the short-latency PLR in the present study is not triggered by melanopsin itself but has already been triggered by rods/cones. The more sluggish recovery profile of pupil responses to white light inside the BS rather than a typical short-latency PLR’s suggests contribution of a longer-lasting process (Fig. 4c).

Our hypothesis, which is based on interactions between ipRGC and rods/cones, seems most parsimonious in explaining all the results in the present study in light of the current physiological knowledge. Gene manipulation that causes ablation of ipRGC cells themselves extinguishes the PLR^30^,^31^, however, it is possible to induce a PLR after the knockout of the gene that encodes the melanopsin photopigment in ipRGCs if the other functions of the ipRGCs are left intact^19^,^30^,^31^. This suggests that ipRGCs, within which melanopsin is expressed, are an essential locus to induce the PLR, not only by activation of melanopsin, but also by signal inputs originating from rods/cones. The present study suggests that the natural BS is actually able to receive light. The new insights into possible contribution of melanopsin inside the BS will be a clue to uncover the biological mechanisms of unconscious vision.

## Methods

### General Methods

#### Observers

This study followed the Declaration of Helsinki guidelines and was approved by the Ethics Committee of the College of Arts and Sciences, the University of Tokyo. The methods were carried out in accordance with the approved guidelines. Informed consent was obtained from all observers. Twelve observers who were naïve to the purpose of the experiment and author KM participated (18–29 years old). All had normal or corrected-to-normal visual acuity.

#### Apparatus

In a dark room, stimuli were displayed on a colour CRT monitor (Mitsubishi Electric RDF223H, 75 Hz, 2.1 arcmin/pixel, 46.5 deg. × 35.7 deg.) with a refresh rate of 75 Hz, controlled by a computer (Apple PowerMac G5). Each observer’s head was fixed with the help of a chin rest. The viewing distance was set at 52 cm. The left eye of each observer was completely occluded by an opaque acrylic cup. Pupil diameter changes and gaze positions of the right eye were recorded with an eye tracker (SR Research, Eyelink2) at 250 Hz. For the experiments shown in Fig. 5, stimuli were displayed on an LCD monitor (VPixx Technologies VIEWPixx, 1.5 arcmin/pixel, 49.0 deg. × 31.7 deg.) with a refresh rate of 120 Hz.

#### Mapping of the right-eye BS

Prior to the main experiment, the BS of the right eye of each observer was approximated by an oval shape in the screen coordinates^32^. First, an oval stimulus was presented to the right of a fixation point (FP) located at the centre of the screen, and each observer adjusted the location and size of the oval with a computer mouse and keys for rough adjustment. Next, the oval stimulus was extinguished, and a small spotlight (6.3 arcmin in diameter) was displayed near the oval border. Observers pressed a key as soon as they detected the spot. Fifty-six spots (seven distances from the border × eight radial directions from the centre of the oval) were displayed in random order during each session. This spotlight perimetry around the border was repeated for at least eight sessions. The positions that corresponded to a 50% detection rate were defined as the detection threshold points, and the adjacent points were connected by straight lines to shape a polygon. The largest oval that just inscribed it was geometrically determined and was used in the main experiments as an estimate of the BS.

#### Measurement of pupil diameter

The pupil diameter of the right eye was measured. Observers fixated at the central FP throughout each trial, and were instructed to perform the attentional fixation task described below to ensure the maintenance of gaze fixation and stable attention to the FP. At the beginning of each trial, a target consisting of two small concentric circles was displayed at the FP. After fixation on the target for 500 ms, the inner circle changed to a Landolt C, broken on one of the four sides (left, up, right, or down), for 50 ms. The observer had to detect the broken side of it. Immediately after this change, a light stimulus was displayed for 80 ms, which is known to trigger a sufficiently large PLR^15^, though much smaller than maximally attainable contraction. A beep sound was delivered 700 ms after the light stimulation, and the observer reported the broken side of the Landolt C by pressing one of computer keys. The beep sound and button press action had no influence on pupillary responses. There was a random inter-trial interval of 5.5 ± 1.5 s, during which the screen remained dark (except for the FP) to help the observers recover from pupil contraction.

### Task design

#### Relation between PLR and perceptual filling-in

We used three stimulus shapes: a “small disk”, which was the estimated BS shape, a “large disk”, which was a similar shape to the estimated BS but with an area twice as large, and a “ring” stimulus, which was made by subtracting the region of the small disk from that of the large disk. We used red and white stimuli, the luminance of which were 18.1 cd/m^2^ and 31.0 cd/m^2^, respectively. The background was a uniform grey of 15.5 cd/m^2^. The procedure involved three stimulus location conditions. In the “test” condition, the stimuli were centred at the centre of the BS. Thus, the small disk covered the inside of the BS and was invisible, and both the large disk and the ring stimulated the area surrounding the BS in the same way and were perceived as a large completed surface (Fig. 1a, upper left). The stimuli were displayed outside the BS under the following two control conditions for the white light. In the “eccentricity-control” condition, the stimuli were presented at the same eccentricity, but shifted above the BS. In the “meridian-control” condition, the stimuli were shifted to the left of the BS. In both conditions, the stimuli and the BS never overlapped; the large disks were located such that their borders just touched the border of the large disk for the test condition (Fig. 1a, upper right). Each observer experienced one of these two control conditions. As the results were the same, we merged the data from the two conditions and considered these data as the “control” condition. For the red light, the “eccentricity-control” condition was used for each observer.

#### Influences of actual percept on the PLR

In some sessions, we examined whether the PLR was related to the physical stimulus shape or to the perceived shape. Either a large white disk or a white ring was displayed at the BS in random order. In addition to conducting the attentional fixation task, observers were requested to judge whether the presented figure was a complete disk by pressing a computer key.

#### Influences of unseen light at the BS on the PLR

In some sessions, we delivered a small disk stimulus inside the BS simultaneously with an increase in background luminance. During the light stimulus presentation inside the BS, the luminance of the whole background of the screen increased from 15.5 cd/m^2^ to 16.7 cd/m^2^, except within the region corresponding to the large disk (Fig. 4a). In the “BS null” condition, only the background luminance changed transiently. In the “BS red” condition, a small red disk was simultaneously presented in the BS. In the “BS white” condition, a small white disk was simultaneously presented in the BS.

In other sessions for one observer, we simultaneously delivered a small disk stimulus inside the BS with its wavelength spectral distribution restricted by a blue (Fuji Film BPB-45) or green (Fuji Film BPB-53) band-pass filter. During the stimulus presentation inside the BS, the luminance of the whole background of the screen increased from 1.5 cd/m^2^ to 1.88 cd/m^2^, except within the region corresponding to the large disk (Fig. 5a). In the “BS null” condition, only the background luminance changed transiently. In the “BS stim.” condition, a small blue or green disk was simultaneously presented in the BS. In the “BS only” condition, a small blue or green disk only was presented in the BS, without an increase in background luminance. The luminance of the blue and green stimuli were adjusted so that they would induce a comparable PLR if they were presented outside the BS at the same eccentricity (blue: 1.48 cd/m^2^; green: 7.42 cd/m^2^; see Supplementary Fig. S3). In other sessions (Fig. 5e), we also illuminated only the left (“BS stim. L” condition) or right (“BS stim. R” condition) half of the BS, simultaneously with an increase in background luminance within the left visual hemifield (24.5 deg. × 31.7 deg.). We collected at least 48 trials in each stimulus condition in each experiment.

### Data Analysis

#### Quantification of the PLR

The pupil diameter was normalized for each trial as a fraction of the baseline diameter averaged over the 500 ms before stimulus onset. Observers with a change in pupil diameter less than 0.5% of the baseline were excluded from the analysis. The number of observers who passed this criterion is shown in the corresponding figure legend, and ranged from 71% to 100%. However, inclusion of the observers with poor contraction in the analysis did not change our main conclusions. Trials including blinks within 1 s of the stimulus onset, trials with bad fixation, and trials with incorrect responses for the attentional fixation task were removed. Trials were also excluded if the minimum pupil diameter was more than ±2 s.d. of the average minimum pupil diameter across all trials under each condition.

We measured the time course of the pupil diameter in at least 30 trials for each condition in each observer in each experiment. Typical average time courses of pupil diameter and their s.e.m. across trials from one observer are shown in Fig. 2a. For each condition, we averaged the time course across all trials within an observer and identified the time at which the pupil diameter became minimal. Pupil diameter changes from the baseline in each trial were averaged across an interval of ±100 ms centred at this time (Fig. 1b). This value was then averaged across all trials. Finally the PLR after z-transformation within each observer was compared across stimulus conditions. The velocity was calculated as the difference between each pair of neighbouring time points in the averaged time course. The maximum velocity of pupil contraction after z-transformation within each observer was also compared across conditions.

We calculated the second-order derivative (acceleration) of the average time course for each observer under each condition and the time from the stimulus onset to the time of the minimum (i.e., most negative) acceleration, which was determined as the light reflex latency^33^.

To evaluate the contribution of white light illumination inside the BS to the PLR, we calculated the difference in the time course of pupil diameter (Δdiameter) between presentation of a large disk and presentation of a ring on the BS (BS[L disk - ring]) and compared this to Δdiameter between presentation of a large disk and presentation of a ring outside the BS (Control[L disk - ring]). We removed unstable trials, including those with blinks and bad fixation within 2.4 s of the stimulus onset, and calculated the average time course of pupil diameter in each stimulus condition in each observer. We compared the latency of the peak Δdiameter (i.e., the greatest difference) between the BS and Control conditions across observers. We separated each Δdiameter trace into the following three periods and compared the traces between the BS and Control conditions within each period: 1) contraction period (from the stimulus onset to the peak of the control trace [0–564 ms]), 2) recovery period I (from the peak of the control traces to the time at which the traces had recovered to zero [568–1496 ms]), and 3) recovery period II (the 0.5 s after the control traces had recovered to zero [1500–2000 ms]).

## Additional Information

**How to cite this article**: Miyamoto, K. and Murakami, I. Pupillary light reflex to light inside the natural blind spot. *Sci. Rep.* **5**, 11862; doi: 10.1038/srep11862 (2015).

## Supplementary Material

Supplementary Information
