# Supplementary material for: Pupillary light reflex to light inside the natural blind spot
Source: Sci Rep. 2015 Jun 26;5:11862. doi: 10.1038/srep11862 (PMC4481772; doi:10.1038/srep11862)
Supplement: Supplementary Information [file srep11862-s1.pdf]

Supplementary information

Pupillary light reflex to light inside the natural blind spot

Kentaro Miyamoto [1,2] and Ikuya Murakami [1,3,*]

1 Department of Life Sciences, The University of Tokyo, Tokyo 153-8902, Japan

2 Department of Physiology, The University of Tokyo School of Medicine, Tokyo 113-0033, Japan

3 Department of Psychology, The University of Tokyo, Tokyo 113-0033, Japan

*Contact information:
Ikuya Murakami
ikuya@l.u-tokyo.ac.jp

Supplementary Figures

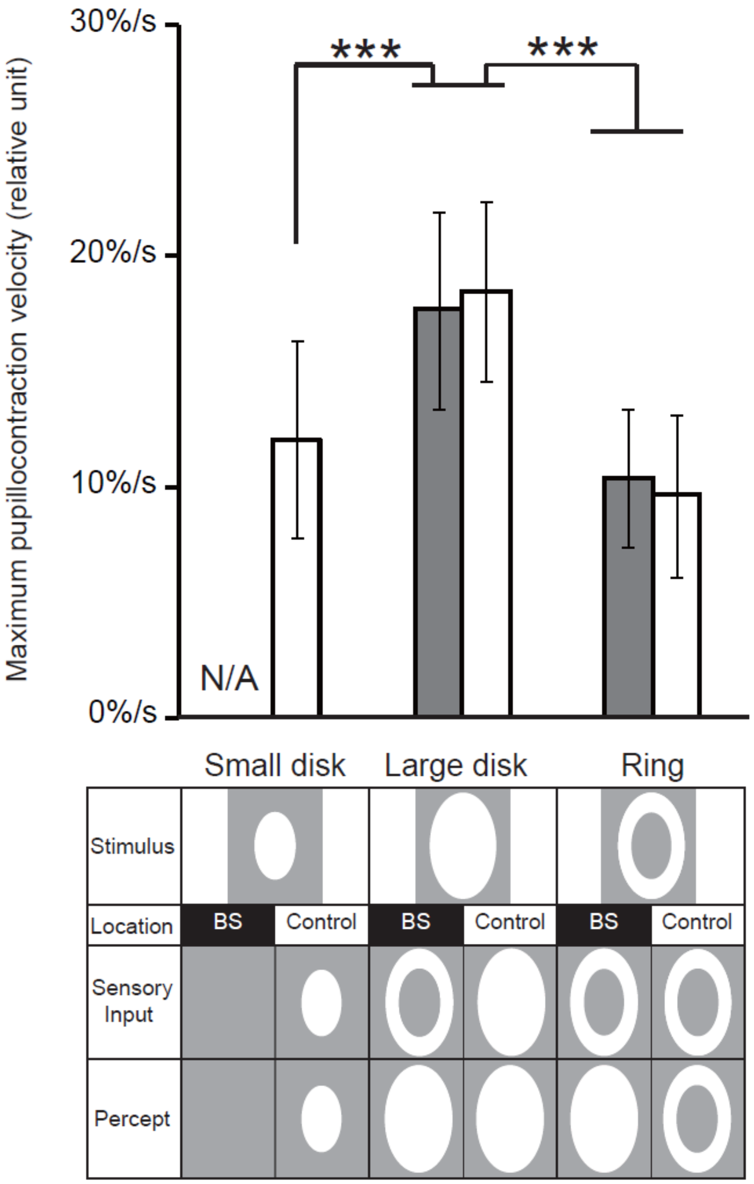

Supplementary Figure S1. Maximum velocity of pupillary light reflex (PLR) time course in response to white light stimuli with perceptual filling-in.

Maximum velocity of the PLR time course (mean ± 1 s.e.m.) across stimulus conditions for white light [N = 10 observers]. *** $p < 0.005$ (Bonferroni-corrected).

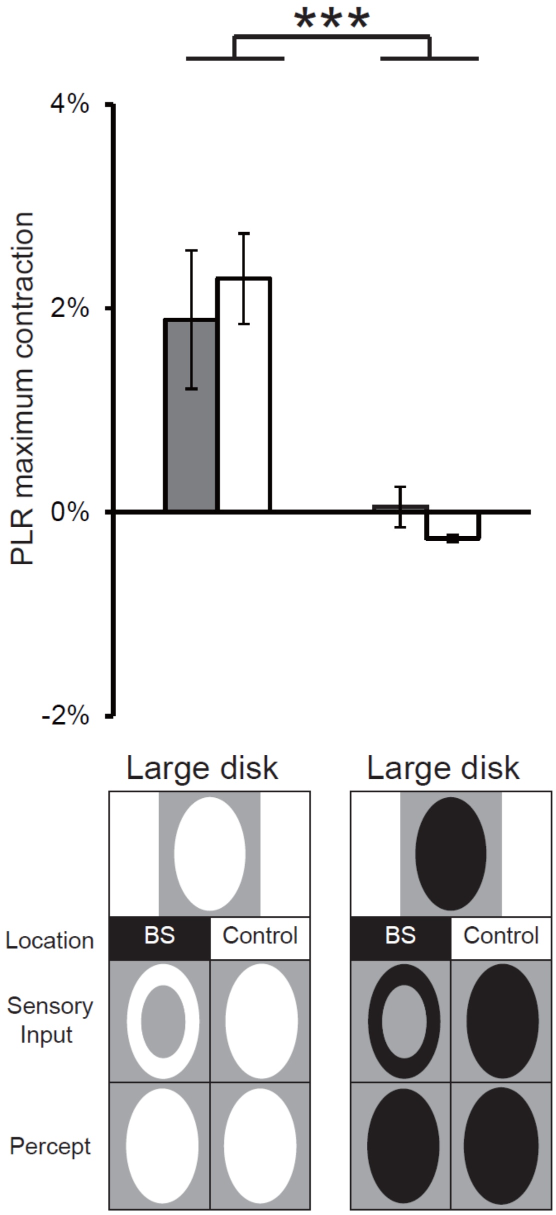

Supplementary Figure S2. Pupillary light reflex (PLR) in response to spatial pattern changes by presentation of stimuli.

PLR size (mean ± 1 s.e.m.) for stimulus presentation with an increment (31.0 cd/m^2) and decrement (0.0 cd/m^2) of light intensity from a uniform grey background (15.5 cd/m^2) [N = 3 observers]. *** $p < 0.005$.

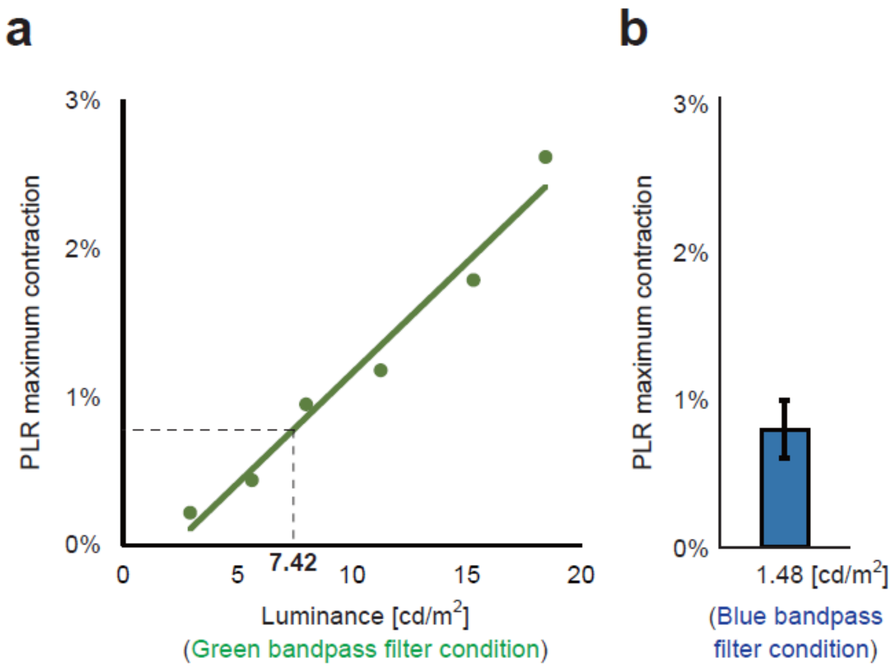

Supplementary Figure S3. The luminance of green and blue stimuli adjusted to induce comparable pupillary light reflex (PLR) outside the blind spot (BS).

The luminance of the blue and green stimuli presented inside the BS (see Fig. 5a) was adjusted so that they would induce a comparable PLR if they were presented outside the BS at the same eccentricity. (**a**) green: 7.42 cd/ m^2. (**b**) blue: 1.48 cd/ m^2.

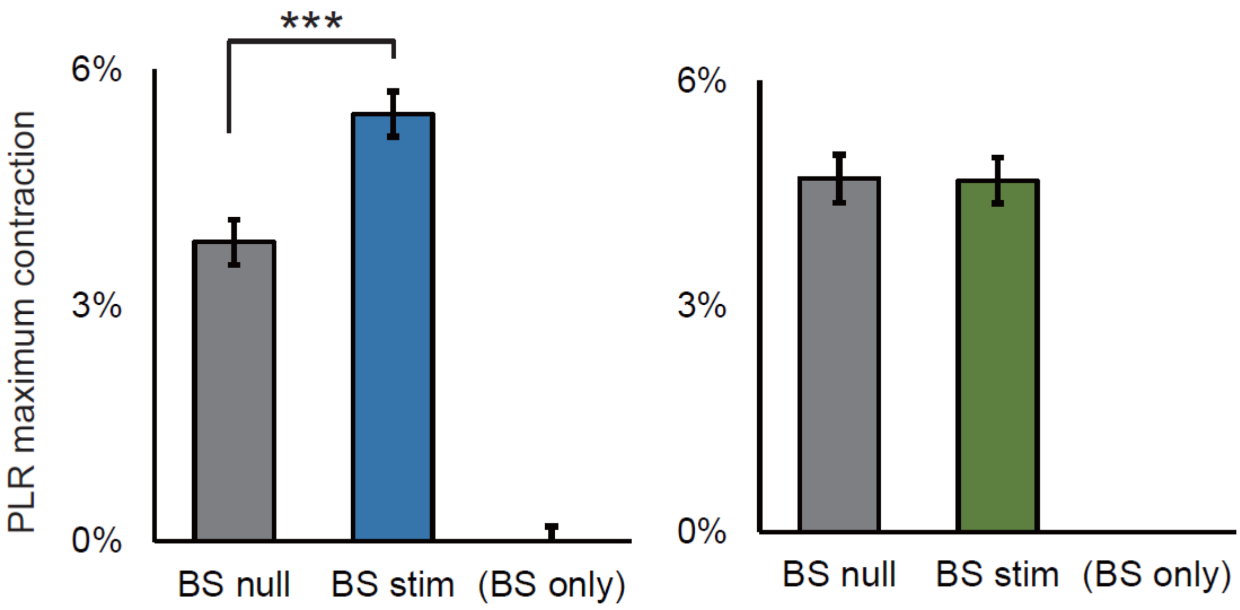

Supplementary Figure S4. Pupillary light reflex (PLR) in response to light stimulation confined to the region around the centre of the blind spot (BS).

Results of experiments identical to that illustrated in Fig. 5a, but the small disk was shrunk to 80% of the estimated size of the BS, and the luminance of both the blue and green stimuli was set at 2.96 cd/ m^2. PLR size (mean ± 1 s.e.m.) in each condition in the experiment using the blue disk (left panel) and green disk (right panel). PLR size in "BS only" condition using the green disk: −0.71 ± 0.12 %. *** $p < 0.005$ (Bonferroni-corrected). In both experiments, the PLR sizes in the "BS null" and "BS stim." conditions were significantly greater than the PLR size in the "BS only" condition ($p < 10^{-5}$, Bonferroni-corrected).
